# Obstructive sleep apnea: a review for the orthodontist

**DOI:** 10.1590/2177-6709.28.1.e23spe1

**Published:** 2023-04-14

**Authors:** Juan Martin PALOMO, Vicente Dias PICCOLI, Luciane Macedo de MENEZES

**Affiliations:** 1 Case Western Reserve University, School of Dental Medicine, Department of Orthodontics (Cleveland/OH, USA).; 2 Pontifícia Universidade Católica do Estado do Rio Grande do Sul, Faculdade de Odontologia (Porto Alegre/RS, Brazil).

**Keywords:** Obstructive sleep apnea, Sleep apnea syndromes, Airway obstruction, Orthodontics

## Abstract

**Introduction:**

Obstructive sleep apnea (OSA) affects an important part of the population and is characterized by recurrent total or partial obstruction of the upper airway (UA) during sleep, negatively affecting the quality of life of patients in the short and long terms, and constituting an important public health problem for the society. The field of expertise of orthodontists is closely related to the UA, placing them in a strategic position to diagnose air passage failures and intervene when necessary. Orthodontists, as health professionals, must know how to recognize respiratory problems and manage them appropriately, when indicated.

**Objective:**

Thus, this paper aims to review and critically evaluate the related literature, to provide orthodontists with updated knowledge on the diagnosis and therapy related to OSA. Science and technology are constantly evolving; thus, the literature was also reviewed considering new technologies available in consumer-targeted applications and devices for the diagnosis, monitoring, and treatment of sleep-disordered breathing.

## RESUMO

**Introdução:**

A apneia obstrutiva do sono (AOS) afeta uma importante parcela da população e caracteriza-se pela obstrução total ou parcial recorrente da via aérea superior (VAS) durante o sono, o que afeta negativamente a qualidade de vida dos pacientes no curto prazo e no longo prazo, e constitui importante problema de saúde pública para a sociedade. A área de atuação do ortodontista está em íntima relação com a VAS, o que o coloca em uma posição estratégica para diagnosticar falhas na passagem de ar e intervir quando necessário. É imperativo que o ortodontista, como profissional da saúde, saiba reconhecer problemas respiratórios e manejá-los de maneira apropriada, quando indicado.

**Objetivo:**

O objetivo desse artigo é revisar e avaliar criticamente a literatura pertinente, para proporcionar ao ortodontista conhecimento atualizado sobre o diagnóstico e terapêutica relacionados à AOS. Ciência e tecnologia estão em constante evolução; portanto, a literatura também foi revisada considerando as novas tecnologias disponíveis em aplicativos e dispositivos direcionados aos consumidores para o diagnóstico, monitoramento e tratamento dos distúrbios respiratórios do sono.

## INTRODUCTION

Breathing is a simple and essential act for the organism, performed millions of times throughout life. The act of breathing takes the oxygen from the air to the cells, and helps to eliminate carbon dioxide, which is a vital process for the metabolic activities of human beings. Any change in this process can trigger health problems. The so-called sleep-disordered breathing (SDB) involves all airflow abnormalities during sleep, from primary snoring without hypoxia, with or without sleep interruption, to obstructive sleep apnea (OSA) with complete blockage of the airway and interruption of the air passage.^1^ The number of people affected by OSA is varied, with values ​​estimated at 14% of men and 5% of women^2^ to much higher numbers, such as 34% of men and 17% of women.^3^ It is believed that the prevalence of OSA in the population may be even higher, since many patients do not have an adequate diagnosis. Respiratory problems can also affect the child population, with adverse manifestations and consequences.^1^ Among adults, daytime sleepiness is among the most common symptoms; however, many patients with OSA are asymptomatic. Individuals may experience serious lifelong consequences such as cardiorespiratory failure, hypertension, type 2 diabetes mellitus, and neurophysiological deficits associated with increases in mortality rate. Thus, OSA is an important public health problem for society, and should be investigated to allow the adoption of preventive measures. Orthodontists intervene in the craniofacial complex and thus they can assist in the recognition of SDB, contributing to the identification of dentofacial components involved and, in some cases, to the treatment of OSA, in association with the physician and team.^2^ Even though in the past only complete obstruction of the air passage during sleep was considered harmful, currently any form of SDB should be considered worrisome since even the least severe of these conditions can have important consequences on the quality of life and general health of individuals. 

Many health professionals are unaware of the role of Dentistry, especially Orthodontics, in the interdisciplinary management of SDB. This review article aims to compile relevant evidence-based information on the role of orthodontists in risk assessment and assistance in the treatment of obstructive sleep apnea, acting in an integrated and multidisciplinary manner to maximize benefits and minimize the side effects. The scientific literature was also consulted in the search for new technologies, such as consumer-oriented applications and devices for the diagnosis, monitoring, and treatment of SDB.

## OBSTRUCTIVE SLEEP APNEA (OSA)

Obstructive apnea is the most common type of sleep-disordered breathing. It is characterized by recurrent episodes of partial or total collapse of the upper airway during sleep, leading to reduced (hypopnea) or absent (apnea) airflow lasting at least 10 seconds and associated with cortical excitation or a drop in blood oxygen saturation.^3^ Sleep is essential for life. Thus, the lack of restful sleep can cause difficulties in attention and concentration, reduction in quality of life and productivity, besides favoring the occurrence of accidents, coronary artery disease, heart failure, high blood pressure, obesity, type II diabetes, memory deficits, stroke, and depression.^4^

**Figure 1:**
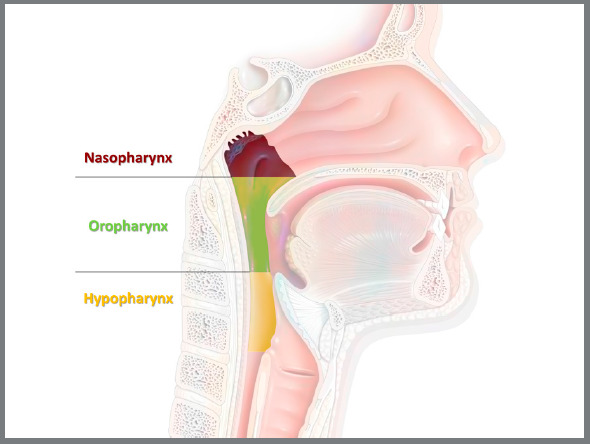
Upper airway, with areas of the nasopharynx (between skull base and hard palate), oropharynx (soft palate to the upper border of the epiglottis), and hypopharynx or laryngopharynx (from the tongue base to the lower border of the cricoid cartilage).

The respiratory system is responsible for the absorption of oxygen from the air and the elimination of carbon dioxide from cells, being formed by the airway (conductive portion) and lungs (respiratory portion). The respiratory tract is the entire path crossed by the air from the nose to the lungs and is divided into upper airway (UA) and lower airway. The UA comprises the nasal cavity, pharynx, and larynx and thus is more relevant for orthodontists because it is close to their area of expertise. The pharynx is a muscular membranous tube that extends from the skull base to the lower border of the cricoid cartilage.^5^ The pharynx is divided into three parts (Fig 1): nasopharynx (skull base to the hard palate), oropharynx (soft palate to the upper border of the epiglottis) and laryngopharynx or hypopharynx (tongue base to the lower border of the cricoid cartilage).^5^ ^,^ ^6^ The velopharynx is part of the oropharynx and is located between the soft palate and the posterior pharyngeal wall. The pharyngeal tonsils (adenoids) are aggregates of lymphoid tissue located on the roof of the posterior nasopharyngeal wall. The palatine tonsils are located between the palatine arches bilaterally in the oropharynx.^5^

The UA is responsible for swallowing, speech and breathing. The accomplishment of these tasks depends on the complex interaction of more than 20 muscles that surround the UA: muscles that regulate the soft palate position (levator and tensor palatini), the tongue (genioglossus, geniohyoid, hypoglossus, and styloglossus), muscles attached to the hyoid bone (hypoglossus, genioglossus, digastric, geniohyoid and sternohyoid) and muscles of the posterolateral pharyngeal walls (palatoglossus and pharyngeal constrictors).^7^ ^,^ ^8^ These muscles act dilating or constricting the UA lumen. The UA is not a rigid structure (e.g., like the trachea) and is fixed only at its upper (skull base) and lower (cricoid cartilage) ends. The hyoid bone is an important site of attachment for pharyngeal muscles, yet it does not have rigid articulation with other bony structures. Therefore, the pharyngeal cross-sectional area depends on the air pressure existing in the upper airway lumen, which can collapse due to different factors.^9^ Contraction of the upper airway dilator muscles (especially the muscles that control the tongue, soft palate, and lateral pharyngeal walls) maintains respiratory patency during inspiration. A drop in the tone of pharyngeal dilator muscles results in airway collapse. Areas of upper airway collapse occur most often in the oropharynx in patients with obstructive sleep apnea (OSA).

The variety and complexity of vibrations and upper airway collapsibility during sleep depend on multiple factors such as sleep stages, muscle tone, body position, head and neck position, and lung volumes. The most common and known sites of obstruction and vibration are located in the soft palate, on the lateral pharyngeal walls, including the tonsils and tongue base. Obstruction at the epiglottic level occurs less frequently but has clinical significance. Many patients with OSA have a multilevel obstruction, with collapse in the retropalatal and retrolingual regions. Single-level obstruction is more common in patients with mild OSA, while multilevel obstruction is more characteristic in severe OSA, which is often the reason for the severity of OSA.^10^

Some clinical signs and symptoms that may be associated with OSA include excessive sleepiness, fatigue, non-restorative sleep, nocturnal snoring, interruption in breathing, choking during sleep, nocturnal diuresis (at least twice during the night), nocturnal gastroesophageal reflux and headache when waking up.^3^

## OSA DIAGNOSIS AND RISK ASSESSMENT

The diagnosis and severity of OSA are determined by polysomnography, in which several sleep and respiratory parameters are monitored simultaneously. One parameter measured is the apnea-hypopnea index (AHI), which assesses the mean number of apneas and hypopneas per hour of sleep. Apnea is the absence of inspiratory airflow for at least 10 seconds, while hypopnea is a reduction of 30% or more in airflow from baseline associated with a drop in arterial hemoglobin saturation and/or an electroencephalographic awakening.^11^

The severity of adult OSA is classified as mild (5≤AHI<15 events/h), moderate (15≤AHI<30 events/h), or severe (AHI≥30 events/h).^11^ In children, OSA is diagnosed when they have an AHI of 1 or higher. OSA in children can be categorized as mild (1≤AHI<5 events/h), moderate (5≤AHI<10 events/h), or severe (AHI≥10 events/h).^1^ A summary of the values for classifying OSA in adults and children is shown in Table 1. The prevalence of OSA increases with age, being twice as common in men than in women.^3^ OSA is also associated with overweight and obesity.^3^

**Table 1:** Summary of values for classification of obstructive sleep apnea (OSA) in adults and children, according to the apnea-hypopnea index (AHI).

OSA risk	Mild	Moderate	Severe
Child	1 ≤ AHI < 5	5 ≤ AHI < 10	AHI ≥ 10
Adult	5 ≤ AHI < 15	15 ≤ AHI < 30	AHI ≥ 30

Many individuals do not report having sleep disturbances, either because they are asymptomatic or because they do not realize they have the problem. Thus, it is important to apply specific questionnaires (Table 2) to assess the risk of an individual having OSA.^3^ ^,^ ^12^ The Berlin and Stop-Bang questionnaires present high sensitivity for assessing moderate to severe risk for OSA (77 and 90%, respectively),^3^ noting that sensitivity is the test ability to correctly identify individuals who have the disease, while specificity is the test ability to correctly identify individuals who do not have the disease.

**Table 2:** Recommended questionnaires for obstructive sleep apnea risk assessment.

Questionnaire name and year	Target audience	Number of questions	Type of questions	Assessment
PSQ - 2000 Pediatric Sleep Questionnaire	Children (Age 2 to 18)	22	Yes/No/I don’t know	8 or more Yes answers, or 33% of positive answers when some are left blank or marked “I don’t know” - low or high risk for SDB
ESS-CHAD - 2015 Epworth Sleepiness Scale	Children And Adolescents	8	8 situations to assign scores (0-3)	To assess daytime sleepiness Low sensitivity for AOS
ESS - 1991 Epworth Sleepiness Scale	Adults	8	8 situations to assign scores (0-3)	To assess daytime sleepiness Low sensitivity for AOS
BQ - 1999 Berlin Questionnaire	Adults	10	3 categories	High Risk: if there are 2 or more categories where the score is positive.
NOSE - 2004 Nasal Obstruction Symptom Evaluation Scale	Adults	5	5 conditions to assign scores (0-4)	To assess potential nasal obstruction
STOP-Bang questionnaire - 2008	Adults	8	Yes/No questions + data BMI, age, neck circumference and gender	High sensitivity and specificity to diagnose the adult patient with moderate to severe OSA

The orthodontist may perform risk assessment for SDB yet diagnosing SDB is outside the scope of dental professionals. A patient at risk requires referral to a sleep physician for proper diagnosis and a possible referral to the orthodontist for treatment with mandibular advancement devices.^12^ There is no specific type of physical finding on examination for OSA. However, the risk of OSA is doubled for overweight individuals and four times higher in obese patients when compared to individuals without these conditions.^3^ Examination of the upper airway may indicate anatomic abnormalities such as hypertrophied adenoids, macroglossia, or mandibular retrusion, but normal conditions do not rule out the presence of OSA.^3^ If the clinical evaluation indicates any clinical signs or symptoms that may be associated with OSA, specific tests are recommended. The diagnosis of OSA requires patient assessment during sleep. The gold standard diagnosis is laboratory polysomnography (type I), which includes a series of assessments such as electroencephalogram, electrooculogram, electromyography of the chin region, airflow analysis, oxygen saturation, respiratory effort, electrocardiogram/heart rate, body position, and movements during sleep.^12^ Polysomnography is a judicious test and allows the diagnosis of other sleep disorders besides OSA. The disadvantage of this test is the high cost, around 5 times the cost of the so-called home sleep apnea testing (HSAT).^3^

Home testing (HSAT) for OSA has been increasingly used, due to its low cost and practicality. It measures the airflow, respiratory effort, and oxygen saturation, yet sleep and leg movements are not measured. The sensors are applied by the patient at home, following the technical instructions provided. This type of test has high sensitivity and specificity; however, it can provide false-negative results. Home tests (HSAT) are not indicated to evaluate respiratory disorders other than OSA.^3^

Cone-beam computed tomography (CBCT) is a well-accepted oral and maxillofacial diagnostic imaging tool that provides a three-dimensional (3D) view of the hard and soft tissue structures of the head and neck, allowing the operator to examine areas, volumes, and complex hollow structures as the upper airway.^13^ Understanding the CBCT grayscale is fundamental since it is the basis for the segmentation process and surface reconstruction of 3D models, used in upper airway analysis, virtual planning of orthognathic surgery, assessment of impacted teeth, and 3D superimposition.^14^ Airway imaging captures only a static view of a dynamic structure; thus, the minimum cross-sectional area (MCSA) is a very important finding that should always be evaluated when performing airway analysis. This can be detected by the software in an automatic configuration, with volume measurement; or it can be activated by specific features of different software. MCSA is believed to be a more explanatory variable than volume in defining pathological conditions of the airway.^15^ Thus, the association of other diagnostic methods, as well as collaboration with a sleep doctor or otolaryngologist, allows a more comprehensive and necessary assessment for the diagnosis of OSA.^15^ Other complementary exams requested for airway assessment are shown in Table 3, with a description of some of their advantages and limitations.

**Table 3:** Some tests requested to assess the airway, with a description of their advantages and limitations.

EXAM	INDICATION	ADVANTAGE	LIMITATION
Lateral cephalometric radiograph	Provides a 2D evaluation of the profile	Adenoid hypertrophy evaluation	2D representation of a 3D structure. Provides a limited use for UA assessment, as the mediolateral direction in not evaluated
Conventional tomography (CT)	3D evaluation	One of the best imaging modalities for evaluating the nasal cavity and paranasal sinus geometry	High doses of radiation
Cone Beam CT (CBCT)	3D evaluation	Good to evaluate hard tissue structures, good to visualize the airway lumen	No information on muscular tone, susceptibility to collapse or function of the airway. Cannot be used to diagnose OSA alone. There is no direct link between radiographic measures (airway size and volume) with PSG
Magnetic resonance imaging (MRI)	Accurate in measuring the soft tissue lining, fat pad and surrounding structures of the airway in 3D	Good to visualize the airway lumen. No radiation	Metals (fillings and braces) can interfere with the images
Nasoendoscopy	Gold standard for diagnosis of UA obstruction	Direct and functional view of the airway in real time. No radiation	Little opportunity for objective measurement, relies on professional opinion (low interobserver agreement)
Acoustic rhinometry (AR)	Objective method for examining the nasal cavity (evaluates the sound pulse propagation in the nasal cavity by changes in acoustic impedance)	Simple, fast, and noninvasive technique. Clinically useful with very good reliability in the anterior and middle parts of the nasal cavities. No radiation	Reduced accuracy in the posterior part of the nasal cavity
Acoustic pharyngometry	Acoustic reflection technique provides a noninvasive, dynamic assessment of the physiologic behavior, dimensions, and structure of the UA	Useful method to assess OSA. Marketed as a screening method to quickly assess potential sites of UA obstruction and to determine the appropriate treatment. No radiation	Limited accuracy, applicability, and information
Ultrasonography -Ultra sound (US)	Assessment of the UA with no use of ionizing radiation	Simple, fast, and noninvasive technique. Assessment of the UA while in function. No radiation	Evaluation relies on professional knowledge and experience

The use of ultrasound in the detection of risk factors for OSA is not new but has been evolving in the last few years, emerging as a promising technology. The technique is radiation-free, less expensive and the equipment is portable, allowing high accessibility for examination and screening. Initially, ultrasound studies focused mainly on anatomical features, not considering the dynamic aspect of the airway, which may better reflect the influence of neurological control of OSA. Subsequently, studies were conducted to assess the association between OSA severity and pharyngeal parameters using submental ultrasound, besides investigating the accuracy of ultrasound to identify patients with severe OSA. Nowadays, the trend is to provide a risk assessment for OSA based on the collapsibility of the airway.^16^

## TREATMENT MODALITIES OF OSA IN ADULTS

The treatment of OSA must be individualized considering the pathophysiology of the disease, the individual characteristics of patients, and the treatment goals. Therapeutic possibilities include behavioral changes, oropharyngeal exercises, positive air pressure devices (PAP), intraoral appliances, surgeries, and electrical stimulation (Table 4). New technologies are being developed to aid the treatment and monitoring of patients with sleep disorders and will be presented and discussed later.

**Table 4:** Summary of treatment options of OSA in adults, according to the diagnosis.

Treatment options for OSA	
Behavioral changes	Weight loss Aerobic exercises Oropharyngeal exercises Alteration in sleep posture Sleep hygiene measures
Medical and dental appliances	Positive air pressure appliances (PAP) Mandibular advancement devices (MAD)
Surgical procedures	Bariatric surgery (for weight loss) Uvulopalatopharyngoplasty Glossoplasty Maxillomandibular advancement Electric stimulation of the hypoglossal nerve

### 1. BEHAVIORAL CHANGES

Weight loss, implementation of physical exercises, positional adjustment, and sleep hygiene measures are positive and non-invasive changes in the treatment of OSA. 

####  Weight loss 

Obesity is an important risk factor for OSA since the accumulation of fat tissue in the cervical region increases the load on the pharyngeal tissues and impairs respiratory patency.^9^ Weight reduction decreases the load on the pharyngeal tissues and aids in breathing during sleep. Recent RCTs show that lifestyle changes (aerobic exercise, diet, sleep hygiene, and reduction of alcohol and tobacco) aiming at weight loss provide significant improvement in OSA and quality of life.^17^ ^,^ ^18^ In a meta-analysis of 10 RCTs (a total of 702 participants), Edwards et al. observed a significant mean reduction of 8.1 events per hour in the AHI of individuals who included physical exercise and a balanced diet in their routines.^19^

####  Exercises 

Aerobic physical exercises can contribute to weight loss, with positive aspects on cardiovascular and metabolic conditions. However, they are not always easy to adhere to, especially for individuals who already have significant cardiopulmonary diseases.^3^

Oropharyngeal exercises are a non-invasive and low-cost alternative for the treatment of primary snoring and mild to moderate OSA. The dilator muscles of the upper airway are essential for the maintenance of respiratory patency during sleep.^20^ Some patients may have flaccidity of the oropharyngeal muscles. This hypotonia is related to the pathophysiology of OSA in these individuals and deserves therapeutic attention.

Oropharyngeal exercises are derived from speech therapy (myofunctional therapy) and include exercises for the mouth and neck region. They include isometric (continuous) and isotonic (intermittent) exercises aimed at the tongue, soft palate, and pharyngeal lateral walls.^20^ ^,^ ^21^ Oropharyngeal exercises have recently been successfully used in the treatment to reduce the severity of OSA, and are focused on improving the strength, force, and coordination of upper airway muscles by repeatedly moving the tongue base back and forth.^22^ It is believed that strengthening the oropharyngeal muscles, through daily exercises, could prevent the upper airway collapse. The main problem with this therapy is the lack of long-term patient compliance, which is only around 10%. The use of applications associated with smartphones can improve the patient’s adherence to exercises and training.^22^

Guimarães et al.^20^ conducted a randomized clinical study on 31 patients with moderate OSA. The authors concluded that individuals who received oropharyngeal exercise therapy for three months showed significant improvements in disease severity and symptoms compared to the control group.^20^ Another randomized clinical trial obtained similar results in patients with primary snoring, indicating oropharyngeal exercises for these individuals.^23^ To maintain positive results, the patients should include exercises into their daily routines in the long term, which can cause problems related to treatment adherence.^20^ ^,^ ^23^ Oropharyngeal exercises should be considered adjunct treatments in the treatment of OSA and not a substitute for CPAP, for example.^21^ ^,^ ^24^ Treatment with oropharyngeal exercises should be conducted by speech-language therapists with specific training for sleep disorders.

####  Positional changes during sleep 

Positional changes during sleep also influence airway patency. Changing from supine to lateral decubitus is important since it expands the upper airway lumen, especially the retroglossal space.^25^ In patients diagnosed with positional OSA, this is the first measure to be taken. This can be aided by the use of pillows or devices that keep the individual in a lateral position during sleep.^3^ Some smartphone apps include position detection and vibrating alarm functions. In this case, the smartphone should be attached to the chest to recognize the patient’s position, triggering a vibrating alarm when in a supine position. This encourages the patient to change position and the vibration stops as soon as the position is changed.^22^ Even though effective, positional changes should be considered complementary therapies in the treatment of patients diagnosed with OSA.

####  Sleep hygiene 

The term “sleep hygiene” refers to the different positive habits that can be implemented to promote an adequate night of sleep, including changes in lifestyle (physical exercise, diet, substance, and medication use) and the environment where the individual sleeps (lighting, noise, temperature, television, cell phone, etc.).^26^ The objective of sleep hygiene is to identify factors that can be changed to optimize nocturnal sleep. Some sleep hygiene habits proposed by the American Sleep Association include:^27^ organizing the bedroom, so that it is peaceful and relaxing; limiting exposure to bright light at night; turning off electronic devices at least 30 minutes before bed; avoiding eating large meals before bed; exercising regularly and maintaining a healthy diet; avoiding caffeine intake in the afternoon or evening; avoid drinking alcohol before bedtime; reducing fluid intake before bedtime. Though important, the American Sleep Association recommends that sleep hygiene measures should be implemented in combination with other therapeutic actions, and never as an isolated approach.^26^

### 2. MEDICAL AND DENTAL APPLIANCES

####  Positive air pressure devices (PAP) 

The positive air pressure devices (PAPs) prevent the airway from collapsing by generating a flow of pressurized air through a mask fitted to the mouth, nose, or both. The treatment of OSA with PAP is extremely effective and has high-quality scientific evidence.^28^ ^,^ ^29^ PAP devices work by generating positive airway pressure at the pharyngeal level, preventing airway collapse, and eliminating snoring, hypopnea, and obstruction events.^22^ PAPs are the reference therapy in the treatment of OSA, central sleep apnea, and chronic hypoventilation, regardless of the severity of the disease.^24^ ^,^ ^29^ The different PAP types include continuous positive pressure (CPAP), bilevel positive pressure (BPAP), and self-adjusting positive pressure (APAP). The treatment of OSA with PAP is under the responsibility of medical doctors and thus should not be indicated or conducted by dental professionals.

Though highly effective in the treatment of OSA, a considerable number of patients are refractory to treatment with PAP.^30^ ^,^ ^31^ A recent paper evaluated adherence to CPAP in 789,260 individuals and found high variability between different groups of patients, from 51.3% in women aged 18 to 30 years to 80.6% in men aged 71 to 80 years.^32^ Patients with less severe OSA have poor adherence to the use of CPAP and are at increased risk of OSA treatment failure.^30^

####  Intraoral appliances 

**Figure 2:**
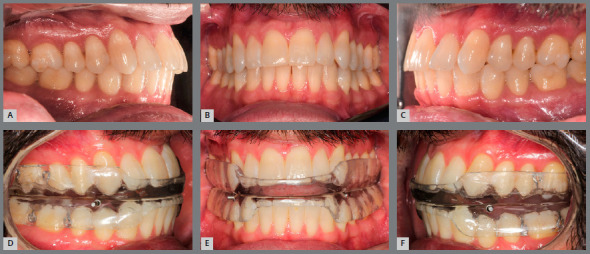
A, B, C) Intraoral view of a patient in maximum intercuspation. D, E, F) The same patient using a mandibular advancement device.

Mandibular advancement devices (MADs) are intraoral appliances that expand the upper airway and decrease its possibility of collapse by projecting the mandible forward during sleep. The patient must place the MAD only during sleep and remove it upon waking. The effects of MAD therapy include: 1) forward traction of the soft palate, tongue, and hyoid bone;^33^ 2) increase in velopharyngeal airway lateral dimension;^34^ 3) stimulation of the upper airway dilator muscles.^35^ ^,^ ^36^ Figure 2 demonstrates the inter-arch relationship of a patient in maximum intercuspation (Fig 2A, 2B, and 2C) and with the use of a MAD (Fig 2D, 2E, and 2F) for the treatment of OSA.

MADs are indicated for the treatment of primary snoring and mild to moderate OSA. Patients demonstrate a high tolerance to the use of MADs and therefore these devices are also indicated in the treatment of severe OSA in patients who cannot stand the use of CPAP.^24^ ^,^ ^37^ ^,^ ^38^ In patients with mild or moderate OSA, MAD reduces the severity of OSA, and excessive daytime sleepiness, and improves the quality of life,^39^ besides reducing the amount and intensity of snoring events.

The definition of success in MAD therapy varies. The treatment is considered effective when it reduces the AHI by at least 50% or when the degree of severity of OSA is reduced (from moderate to mild, for example).^33^ ^,^ ^39^ Sutherland et al.^40^ reported nearly 70% of success in treating OSA with MADs in 425 patients, regardless of demographic and anthropometric factors. In a review published in 2012, Marklund et al.^39^ found a mean reduction of 55% in the AHI of patients treated with MADs. Therefore, MADs do not completely solve OSA, but they reduce the AHI to mild levels and increase oxygen saturation during sleep.^33^

**Figure 3:**
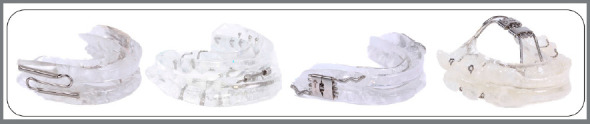
Different mandibular advancement devices (individualized bi-blocks made of rigid acrylic plates that allow titration).

There are many types of MADs with different characteristics. They may be prefabricated or customized, monoblock (non-titratable) or bi-block (titratable). Titration is a jargon inherited from the medical field referring to the adjustment of CPAPs and is used to refer to the possibility of a progressive increase in mandibular advancement by the dentist. Titratable devices are superior to non-titratable devices, especially in the treatment of moderate and severe OSA.^33^ ^,^ ^41^ A small mandibular advancement produces a less than ideal result, while a large mandibular advancement can increase discomfort and side effects of MAD therapy. Customized devices are more comfortable and effective than prefabricated devices. Thus, custom manufacturing and the possibility of titration are important factors in achieving adequate results in the treatment of OSA with MADs.^33^ ^,^ ^39^ MADs are tooth-borne appliances; thus, fabrication with rigid acrylic plates is preferred over acetate plates, which may reduce the comfort yet increase anchorage and minimize the long-term dentoalveolar changes. Figure 3 shows some types of MADs with different characteristics: bi-block and customized appliances, with rigid acrylic plates connected by devices that allow titration. It should be clear that these are just a few examples, and several other types of MADs have been described in the literature. The professionals should define the most appropriate type of MAD for the treatment of their patients.

The main immediate side effects of the use of MADs are changes in salivation (dry mouth or hypersalivation), muscle and temporomandibular joint discomfort, and bite changes.^38^ The patients should be instructed about these possible changes and require an adaptation period (usually two to three months). Patients with temporomandibular joint problems should be evaluated by a specialist in temporomandibular disorder (TMD) and orofacial pain before initiating the use of MAD. However, MADs do not represent a risk factor for the development of TMD signs and symptoms and do not seem to exacerbate preexisting TMD problems.^42^ In a 5-year longitudinal study, Martinez-Gomis et al.^43^ did not observe significant increase in the prevalence of TMD in patients under treatment with MADs.

In a longitudinal review published in 2020, Venema et al.^44^ reported that both MAD and CPAP therapy resulted in positive outcomes after 10 years of follow-up. Therefore, dental professionals should know how to manage the possible long-term side effects of MAD therapy. Expected long-term side effects include the projection of the lower incisors and retroclination of upper incisors, with consequent reduction of overjet and overbite.^45^ ^-^ ^47^ The intensity of these side effects varies between patients. Low-intensity vertical skeletal effects (clockwise mandibular rotation) may also occur in the long term.^46^ ^,^ ^47^ A recent systematic review with meta-analysis^47^ on the long-term effects of MADs revealed an increase of 1.54±0.16º in lower incisor proclination, reduction of 0.89±0.04 mm in overjet and 0.68±0.04 mm in overbite, downward and forward mandibular rotation and reduction in SNA angle of 0.06±0.03º. 

Most of these changes are clinically insignificant, but patients with initially reduced overjet and overbite should be followed carefully. In individuals with significant occlusal changes, the maintenance or discontinuation of MAD should be discussed with the patient to allow correction of tooth positioning. This decision should always include the sleep medicine specialist, who may recommend CPAP therapy if orthodontic intervention is needed. The dentist is an important part of a multidisciplinary team in the treatment of OSA, and the American Sleep Association recommends that only trained dentists should treat sleep disorders with oral devices.^48^

### 3. SURGICAL PROCEDURES

CPAP is the standard alternative for patients with moderate and severe OSA. However, adherence to CPAP is difficult, and less than 50% of patients are effectively treated in the long term.^31^ ^,^ ^49^ Surgical procedures in the airway are the option for the treatment of OSA when CPAP and/or oral devices are rejected or achieve insufficient results. The most investigated surgical procedures for adults are uvulopalatopharyngoplasty (UPFP) and maxillomandibular advancement (MMAS).

####  Uvulopalatopharyngoplasty (UPFP) 

UPFP was proposed in 1981 and involves the removal of the uvula and posterior part of the soft palate, increasing the velopharyngeal area.^50^ Currently, UPFP can be performed with the aid of a laser and can be combined with surgeries in other airway sites (multilevel surgery). UPFP presents different results in the literature. A systematic review including a meta-analysis with 435 adult patients concluded that UPFP is effective in treating patients with OSA, yet the effectiveness is reduced in the long term.^51^ The American Academy of Sleep Medicine does not recommend UPFP as the only surgical procedure in the treatment of OSA.^52^

####  Maxillomandibular advancement surgery (MMAS) and other interventions 

MMAS is an invasive surgery that involves advancement and counterclockwise rotation of the facial bones.^53^ For that purpose, Le Fort I osteotomies are performed in the maxilla and ramus sagittal osteotomies in the mandible. MMAS promotes airway enlargement by physically expanding the facial framework, in which the pharyngeal soft tissues and tongue are attached.^54^ MMAS is the most effective surgical modality in the treatment of OSA, and the greater the severity of OSA, the greater the therapeutic benefit.^54^ ^,^ ^55^ The treatment of OSA with MMAS is effective and has good stability. A retrospective study on 25 patients found a significant increase in oropharyngeal space after MMAS, with mild relapse after 10 months of follow-up.^56^ A recent systematic review with meta-analysis indicated the superiority of MMAS over multilevel surgery in the treatment of OSA.^57^ Ideally, orthognathic surgery therapy should be performed under the supervision of an orthodontist to maximize esthetic and functional gains.

Other surgeries may aid the treatment of OSA. Nasal surgeries such as turbinectomy and septoplasty increase respiratory patency and aid the treatment of OSA.^58^ Nasal surgeries can be performed in isolation or combined with orthognathic surgery. It is important to note that the present paper addresses the treatment of OSA in adult patients; thus, adenotonsillectomy (standard treatment for pediatric OSA) was not addressed here. Bariatric surgery may also be considered in the treatment of OSA in obese patients.^59^

####  Electrostimulation of the hypoglossal nerve (surgical electrode implantation) 

The maintenance of upper airway patency is directly related to the action of oropharyngeal muscles, especially the genioglossus. This therapy aims at stimulating the hypoglossal nerve to increase tongue protrusion and stabilize the UA during inspiration.^3^ The treatment of moderate to severe OSA can be performed with hypoglossal nerve stimulation by a surgically implanted neurostimulator.^60^ This therapeutic modality is relatively new and appears to be an interesting option for patients who do not tolerate CPAP.

The neurostimulator device is implanted in the chest, approximately 2 to 4 cm below the collarbone, and features a wire for stimulation and a wire with a respiration sensor. The stimulation wire is adapted to the main branch of the hypoglossal nerve by a horizontal incision in the upper part of the neck at the lower border of the submandibular gland.^60^ The breath sensor wire is adapted by an incision in the fourth intercostal region, where it is inserted using a tunneling technique.^60^ The patient turns on the device before going to sleep and turns it off when awakening, by activating a remote control.

UA electrostimulation is an effective option in the treatment of moderate to severe OSA, with a significant reduction in objective (AHI and oxygen desaturation index) and subjective measures (Epworth sleepiness scale and quality of life questionnaires).^60^ However, it is a high-cost procedure when compared to other alternative therapies. There may be complications such as temporary weakness, tongue pain, or discomfort from the stimulation.^3^

In a systematic review with meta-analysis, Constantino et al. assessed the long-term clinical effects (5-year control) in a total of 350 patients treated with hypoglossal nerve stimulation. The authors observed a high surgical success rate and a mean reduction of 18 points on the AHI and 5.27 points on the Epworth sleepiness scale.^61^ Wang et al.^62^ reported a less optimistic success rate (61%) in 46 patients treated with hypoglossal nerve stimulation, not being able to define a profile of responsiveness between them. Hypoglossal nerve stimulation is considered an effective option in the treatment of moderate to severe OSA in CPAP-intolerant patients. Patients with Down syndrome have a high rate of persistent OSA and may benefit from electrostimulation treatment.^63^

## ORTHODONTIC TREATMENT AND ITS POSSIBLE INFLUENCE ON THE UPPER AIRWAY

The field of expertise of orthodontists is closely related to the upper airway. Next, some orthodontic treatment modalities and their relationship with possible changes in upper airway anatomy and breathing pattern will be discussed. This paper does not aim to review the treatment of OSA and UA changes in children. However, some of the orthodontic treatments reported are conducted specifically in this pediatric population and thus will be pertinently discussed.

### ANTEROPOSTERIOR SKELETAL PATTERN

####  Headgear 

The negative influence of the headgear on the upper airway volume may be assumed, due to maxillary growth restriction. However, Class II treatment with headgear induces greater dentoalveolar than skeletal changes. Kirjavainen and Kirjavainen^64^ reported that treatment with cervical headgear increased the velopharyngeal dimensions yet did not affect the rest of the oropharynx and hypopharynx. Julko et al.^65^ evaluated the cephalograms of 56 children treated with headgear and also did not find negative changes in the UA.

Kang et al.^66^ investigated the immediate effects on the lower arch after distalization of upper molars with the use of headgear in non-growing patients. The mean duration of headgear use was 6.3 months, with an expanded internal arch (2 mm) and force application (load) of 400 to 450 g per side. There was a mean distalization of 4.2±1.6 mm of the upper first molars and a spontaneous increase in intercanine, inter-premolar and intermolar widths in the mandibular arch. The results indicated that distalization of the upper molars with the use of a slightly expanded headgear induced a spontaneous response in the untreated mandibular dentition of non-growing patients. According to the authors, a possible explanation for these changes would be the balance theory, according to which the new tongue positioning would apply pressure, promoting changes in the lower dentition.^66^

It is believed that all interventions that can potentially affect tongue positioning could promote changes in the upper airway since the tongue base and posterior region constitute the anterior wall of the oropharynx. To date, there is no evidence that Class II treatment with headgear decreases the UA and causes respiratory damage.

####  Maxillary protraction 

Maxillary protraction is performed in growing individuals for the treatment of Class III malocclusion. Systematic reviews demonstrate that maxillary protraction with a face mask can increase the UA dimensions.^67^ ^,^ ^68^ Conversely, studies with a control group do not support this statement.^69^ ^,^ ^70^ In a randomized study, Miranda et al.^71^ evaluated CT scans of 35 patients treated with maxillary protraction (hybrid and conventional hyrax) anchored on mini-implants in the mandible. The authors concluded that there was no difference between groups, and maxillary protraction with the two devices can benefit patients with OSA by promoting a volumetric increase in the region of greatest constriction of the upper airway.^71^ These results should be evaluated carefully since the morphological changes of the upper airway should not be confused with improved respiratory function. In a preliminary study published in 2019, Quo et al.^72^ evaluated, using questionnaires and PSG, the influence of bone-anchored maxillary protraction (BAMP) in the treatment of OSA in 15 patients. The authors concluded that maxillary protraction may aid the treatment of OSA by reducing the AHI of patients with midface retrusion.^72^

####  Functional appliances 

El and Palomo^73^ evaluated, by CBCT, the UA volume of 140 patients with different skeletal patterns and concluded that the mandibular retrognathia of individuals with Class II malocclusion decreases the UA in the oropharyngeal region. Therefore, it seems logical to infer that mandibular protraction appliances can benefit the upper airway volume in these patients by promoting the advancement of the tongue, soft palate, and hyoid bone. Ganesh and Tripathi^74^ conducted a scoping review and concluded that fixed functional appliances increase the dimensions of the oropharynx and hypopharynx. It is important to note that studies with different functional appliances were included, and most of the 18 studies evaluated used cephalograms (2D) to measure the UA. Amuk et al.^75^ conducted a prospective randomized study with a Herbst appliance (associated with RME) and also found an increase in oropharyngeal and hypopharyngeal dimensions. A randomized clinical trial published in 2018 compared the short-term effects of a mandibular advancement device (Twin-block) with a placebo device without advancement (control) and showed a significant reduction of 37% in the AHI of children who used the Twin-block.^76^ Studies with longer follow-up periods should be conducted to prove the maintenance of these effects over the years. Despite the possible beneficial effects on the UA, treatment with mandibular advancement devices should be indicated primarily for Class II correction.^2^ Adenotonsillectomy is the gold standard treatment for OSA in children.

### TRANSVERSE SKELETAL PATTERN

####  Rapid maxillary expansion (RME) 

Rapid maxillary expansion (RME) is the standard treatment for the correction of the transverse maxillary deficiency. Increases in skeletal and dental dimensions are unquestionable and are already well reported. Due to the proximity to the oral cavity, the volume and area of the upper airway and nasal cavity can be changed by RME. Immediately, RME promotes displacement of circumaxillary sutures in the three planes of space^77^ and a significant increase in vertical and lateral dimensions of the nasal cavity,^78^ ^,^ ^79^ as well as in the nasopharyngeal volumes and area of greater UA constriction.^79^ El and Palomo^80^ evaluated, by CBCT, the UA volume of 35 patients treated with RME and followed up for to 2 years. The authors observed a significant increase in the volume of the nasal air passage in the UA compared to the control group, while there was no significant difference in the UA volume in the oropharyngeal region.^80^ In 2017, Camacho et al.^81^ performed a meta-analysis with 17 studies (totaling 314 individuals) and demonstrated a 70% reduction in the AHI of children treated with RME. However, the role of RME in the treatment of OSA is debatable. In a recent randomized clinical trial, Magalhães et al.^82^ compared the effects of adenotonsillectomy (AT) and RME on the AHI and volumetric increase in the UA of 39 children with maxillary constriction. The authors reported a greater reduction in AHI and greater volumetric gains in the UA of the group treated with AT, confirming AT as the gold standard therapy in the treatment of pediatric OSA, and concluding that RME is an adjunct in this process.^82^ The American Association of Orthodontics cautions that there is not enough evidence to support the prophylactic indication of RME for the treatment of pediatric OSA; thus, the primary indication should always be the treatment of maxillary constriction.^2^

####  Miniscrew-assisted RME (MARPE) and surgically-assisted RME (MISMARPE and SARPE) 

In skeletally mature patients, RME can be aided by mini-implants (MARPE) or surgery (MISMARPE and SARPE) to surpass the sutural resistance. Yi et al.^83^ found an 8.48% increase in nasopharyngeal volume in 19 individuals (aged 15 to 19 years) with transverse maxillary deficiency treated with MARPE assessed by CBCT. The authors did not find significant differences in oropharyngeal volume and total UA volume.^83^ Li et al.^84^ also found volumetric increase only in the nasal cavity and nasopharynx after treatment of maxillary constriction with MARPE. Brunetto et al.^85^ evaluated 20 patients (>18 years) with transverse maxillary deficiency treated with MARPE and compared them with a control group of 12 untreated patients with matched characteristics.The MARPE-treated group showed significant respiratory improvement, with a mean reduction of 65.3% in AHI and self-reported improvement (Epworth Sleepiness Scale and Quebec Sleep Questionnaire) in daytime sleepiness and quality of life. Despite the positive results, more well-designed studies with long-term follow-up are needed to prove the beneficial effects of MARPE as an auxiliary therapy in the treatment of OSA in adults.^85^

Minimally invasive surgical and miniscrew-assisted rapid palatal expansion (MISMARPE) was recently proposed by Haas Jr et al.^86^ The technique consists of outpatient surgery with an incision in the buccal sulcus (region of the upper right and left lateral incisors) followed by four osteotomies: a subspinal osteotomy, a vertical midline osteotomy, and two horizontal lateral osteotomies (one on each side), without separation from the pterygoid pillar. The advantage of this procedure is that it is effective for the correction of maxillary hypoplasia in adult patients, by an outpatient procedure, without the need for hospitalization or general anesthesia.^86^ Future studies are warranted to evaluate the effects of the MISMARPE technique on the UA and breathing of patients.

Pereira-Filho et al.^87^ evaluated the effects of SARPE on UA volume in 15 patients (30.2 years, ±7.4 years) with transverse maxillary deficiency. No significant differences were found in UA volume and area before and after SARPE; except for the area of smallest transverse constriction, which was inferiorly repositioned. The authors concluded that SARPE cannot be considered in isolation to improve the UA dimensions.^87^ A recent meta-analysis of 10 studies (a total of 257 individuals) evaluated the impact of maxillary expansion with different techniques (MARPE, SARPE, LeFort I with maxillary segmentation, distraction osteogenesis) in nasal breathing.^88^ Although there is a positive result of maxillary expansion in breathing, the authors concluded that there is not enough evidence to support the indication of this procedure as a treatment to improve nasal breathing in adult patients.^88^ Alike in children, the prophylactic maxillary expansion for OSA does not seem to be indicated in adult patients.

### ORTHOGNATHIC SURGERY

Severe maxillomandibular discrepancies can be treated by orthognathic surgery. Currently, software for surgical planning allows greater safety in the treatment outcomes considering facial esthetics, occlusion, and respiratory function. Orthognathic surgery can be performed on only one bone (maxilla or mandible) or both (bimaxillary). Double jaw advancement surgery with counterclockwise rotation of the occlusal plane (MMAS) promotes a lateral and anteroposterior increase in the UA and is effective in the treatment of OSA.^53^ ^,^ ^54^ This procedure is indicated in the treatment of severe OSA, especially in CPAP-refractory patients with great maxillomandibular discrepancies.

Surgical correction of mandibular prognathism promotes esthetic and functional benefits, but it can also promote undesirable effects, such as airspace narrowing, which in association with other factors, such as a reduction in tongue space, can lead to OSA symptoms. Henkin et al.^89^ reported a case in which the patient developed OSA after surgery first procedure with mandibular repositioning, in an adult Class III patient. In this case, the symptoms of OSA were overcome after a new surgical intervention involving glossoplasty and mentoplasty. The patient expressed satisfaction with the esthetic and functional results achieved by treatment, as well as with the improvement in quality of life.^89^ Yavari et al.^90^ used a STOP-BANG questionnaire to prospectively evaluate 30 patients undergoing isolated mandibular repositioning surgery. The authors reported a significantly increased risk of OSA in mandibular setbacks greater than 5 mm, while the risk of OSA was not increased in mandibular setbacks smaller than 5 mm.^90^ Gandedkar et al.^91^ retrospectively evaluated 48 patients undergoing bimaxillary surgery for the treatment of Class III compared with a control group of Class I patients. Despite the decrease in oropharyngeal volume, the authors did not find an increase in the risk of OSA in setbacks from 4 to 8 mm.^91^ Thus, consideration should be given to treating skeletal Class III with double jaw surgery (i.e., mandibular setback associated with maxillary advancement), rather than single jaw mandibular setback surgery.

### TOOTH EXTRACTIONS

Treatments with the extraction of first premolars are conducted to correct the tooth size discrepancy and/or adjust the inclinations of incisors. In cases of anterior retraction with maximum anchorage, the arch length is reduced, which could lead to more posterior tongue positioning with a consequent decrease in UA. 

Maaitah et al.^92^ studied, by cephalograms, the effect of first premolar extractions on the UA of 40 patients with biprotrusion. The authors reported a significant reduction in the length of dental arches and tongue (posterior displacement), yet they did not report changes in UA dimensions.^92^ Other studies also did not find changes in UA size when performing orthodontic treatment with extractions of first premolars in different sagittal malocclusions or different vertical facial patterns.^93^ ^,^ ^94^ In a systematic review published in 2015, Hu et al.^95^ concluded that many studies assessed had methodological flaws (conducted in two-dimensional exams or without direct assessment of respiratory function), and thus there is no evidence that orthodontic treatment with tooth extractions can significantly change the respiratory function and UA size.^95^ The same idea was corroborated by Orabi et al.^96^ in 2021, in a systematic review with meta-analysis. They concluded that there is no strong evidence to support the concept that premolar extractions in bimaxillary protrusion or crowded patients reduce the pharyngeal airway volume or the minimum cross-sectional area.^96^

#### NEW TECHNOLOGIES: AUXILIARY APPS AND EQUIPMENT

The development of smartphones has enabled the emergence of apps and technological devices that are increasingly inserted into our daily lives. Particularly in the health area, there is a growing interest in sleep issues and respiratory disorders. Personal sleep tracking devices are being widely used and becoming increasingly technologically advanced, raising strong interest from researchers and clinicians in their use as an alternative to conventional tests.^97^

In the market, the differences between mobile apps for medical or entertainment purposes are usually unclear.^98^ While some seem to work well, meeting the proposed function, others are imprecise or “immature”, considering their cycle of technological development, not yet being reliable.^22^ The critical point is whether these applications work well enough to provide accurate and reliable data.^97^

The American Academy of Sleep Medicine (AASM) advocates that sleep assessment technology devices (SATD), called “consumer sleep technology (CST)”, must be strictly assessed and approved by the Food and Drug Administration (FDA) if intended to provide a diagnosis and/or treatment. Thus, considering the unknown potential of SATD to measure sleep or assess sleep disorders, these tools should not replace medical assessment.^98^ The use of these technologies for the diagnosis, monitoring, and treatment of SDB is very promising, yet remains in the early stages of development.^22^ So far, these devices can be used to improve doctor-patient interaction, when used properly,^98^ and considerably improve patient adherence to treatment.^22^

In 2018, the AASM published a paper to guide professionals on how to approach patients who are interested in using SATD, recognizing their potential benefits for clinical use; however, considering the lack of scientific validation, most SATD still cannot be used for diagnosis and/or treatment of sleep disorders.^98^ There is great potential for SATD for use in research, being promising for data collection and the use of longitudinal sleep-related data.^98^

**Figure 4:**
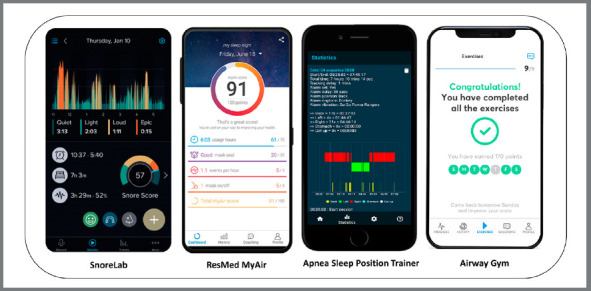
Some sleep assessment technology devices presented in a systematic review,^22^ available on Google Play and iOS platforms.

New technologies require both a learning curve and a review of their reliability.^22^ Thus, Batista et al.^22^ searched for relevant sleep apnea-related apps on the Google Play and Apple App stores. This was associated with a systematic literature review, consulting the Medline, Embase, Web of Science, and Scopus databases for papers published in the scientific literature containing apps or devices used in a clinical environment for the diagnosis or treatment of sleep-disordered breathing.^22^ Among the 161 articles initially selected, 44 were included in the systematic review. A total of 300 smartphone apps were found, with only 10 applications (Table 5) meeting the inclusion criteria associated with published scientific works. The inclusion criteria comprised OSA apps, in English, which could be used in a clinical environment, while applications not relevant to the research scope and/or duplicates, educational apps, advertisements of companies or individuals, or even apps with mandatory use of specific devices, which should be purchased separately, and apps in languages other than English were excluded.^22^ Some apps used in the study are shown in Figure 4.

**Table 5:** Apps presented in a systematic review,^22^ with respective papers and outcomes (found in both platforms iOS and Android, except for those cited in the table). Apps presenting favorable results or compatible with standard diagnostic methods are highlighted in bold.

APP OBJECTIVE AND COMMENTS	APP NAME / PLATAFORM (IF SINGLE)	RESEARCH PAPER RELATED TO THE APP	RESEARCH RESULTS
Sleep monitoring Provides users with a graph detailing the level of wakefulness and light/deep sleep	Sleep time	Bhat S, Ferraris A, Gupta D, Mozafarian M, DeBari VA, Gushway-Henry N, et al. Is there a clinical role for smartphone sleep apps? Comparison of sleep cycle detection by a smartphone application to polysomnography. J Clin Sleep Med. 2015;11.17.	The app was compared to PSG in adults and found a poor correlation between them concerning the sleep efficiency, light sleep, and deep sleep. No correlation was found between the app and PSG sleep latency with a deficiency in detecting wakefulness. Not effective to awaken individuals during light sleep.
Sleep monitoring Accelerometer-based app designed to ease awakening during light sleep periods.	Sleep cycle	Patel P, Kim JY, Brooks LJ. Accuracy of a smartphone applicationin estimating sleep in children. Sleep Breath. 2017;21:505-11.	App data were compared to sleep analysis with PSG in a clinical population of 25 children (2-14 years) with clinical suspicion of OSA. No significant correlation was found between total sleep time and sleep latency compared to PSG. The authors concluded that the app is not yet accurate enough to be used for clinical purposes.
Sleep monitoring Behavioral training response to auditory stimuli estimates sleep onset.	Sleep on Q (iOS)	Scott H, Lack L, Lovato N. A pilot study of a novel smart-phone application for the estimation of sleep onset. J Sleep Res. 2018;27:90-7	Authors found high correspondence between the app and PSG sleep onset. The app tended to overestimate sleep latency. The authors highlight the potential relevance of use for facilitating power naps in the home environment.
Sleep monitoring Monitors and provides feedback on auditory snore activity.	SnoreLab	Stippig A, Hübers U, Emerich M. Apps in sleep medicine. Sleep Breath. 2015;19:411-7.	Authors tested the ability to distinguish between snoring events and other background noise. Results did not correspond to the concurrent validated ApneaLink Plus screening device, which led authors to conclude that reliability and accuracy are insufficient to replace common diagnostic standards.
Adjunct CPAP monitoring Engagement tool that allows patients to track nightly sleep data and empowers patients to stay engaged and compliant with long-term therapy.	ResMed My Air	Woehrle H, Arzt M, Graml A, Fietze I, Young P, Teschler H, et al. Effect of a patient engagement tool on positive airway pressure adherence: analysis of a German healthcare provider database. Sleep Med. 2018;41:20-6	This tool was associated with significant compliance improvement in first-time users receiving CPAP therapy and a significant reduction in air leakage.
Adjunct CPAP monitoring Aims to improve CPAP adherence by a series of text message questions. Patients are asked daily about OSA treatment concerning CPAP use, physical activity, dietary habits, and a weekly recording of bodyweight. Users receive concise recommendations about CPAP use and a healthy lifestyle.	Apnea-Questions (Apnea-Q)	Isetta V, Torres M, González K, Ruiz C, Dalmases M, Embid C,et al. A new mHealth application to support treatment of sleep apnoea patients. J Telemed Telecare. 2017;23:14-8.	Regular users of the app had significantly higher CPAP compliance, with high satisfaction levels for most users.
Orofacial Myofunctional Therapy It is proposed that, by strengthening oropharyngeal muscles by daily exercise, upper airway collapse is avoided.	Airway gym	O’Connor Reina C, Plaza G, Ignacio-Garcia JM, Baptista JardinP, Garcia Iriarte MT, Casado Morente JC, et al. New Health application software based on myofunctional therapy applied to sleep-disordered breathing in non-compliant subjects. SleepSci Pract. 2020;4:1-10	The app provides a permanent record of feedback and accuracy of exercises performed. There was reduction in AHI and in a recent study, authors showed a reduction in AHI and ESS score in 75% of patients using the device after performing daily app exercises for 3 months, compared with a control group.
Orofacial Myofunctional Therapy Users perform 15 minutes of daily voice-activated game play to improve snoring and sleep quality. Users articulate specific phonemes to achieve voice-controlled on-screen objectives.	Snoretech (Android)	Goswami U, Black A, Krohn B, Meyers W, Iber C. Smartphone-based delivery of oropharyngeal exercises for treatment of snoring: a randomized controlled trial. Sleep Breath.2019;23:243-50	Randomized controlled trial with snoring patients showed significant reduction in snoring and ESS after 8-weeks.
Positional obstructive sleep apnea therapy The app aims at position detection (differentiates between prone, back and side positions). Only the preceding night can be displayed	Apnea Sleep Position Trainer	Haas D, Birk R, Maurer JT, Hörmann K, Stuck BA, Sommer JU. Treatment of supine position-related obstructive sleep apnea with smartphone applications. HNO. 2017;65:148-53	Apps were able to prevent positional OSA, being a cost-effective option in the treatment of POSA. Compliance after 6-months was 79.2%.
Positional obstructive sleep apnea therapy In addition to the general functions of position detection and vibration alarm, this app offers a detailed history of position during sleep, of a period.	SomnoPose --- Sleep Position Monitor (iOS)		

This new technology provides affordable, inexpensive, and ongoing home monitoring of OSA, yet it has not yet been adequately assessed and its validation is still questionable. Until accuracy is validated and available, smartphone apps and devices for SDB should be used carefully as an adjunct, rather than as the only method of sleep assessment.^22^ Most SATDs that offer sleep tracking are found in the form of “wearables” that are worn on the wrist or other body areas such as fingers, head, and torso. Conversely, several companies have developed non-wearable devices that are placed close to the user to track sleep using remote sensing of physiological and behavioral signals.^97^ The use of these new technologies for the diagnosis, monitoring, and treatment of SDB is promising but is still in the early stages of development. Smartphone apps and linked devices offer affordable, low-cost, continuous data monitoring at home. However, without proper testing and validation, they can be unreliable.^22^ Peer review, transparent calculations and algorithms, and validation of data used in these technologies can reduce patient risks (such as false positives and negatives), increase clinical confidence, and improve the use of standardized metrics and practices for devices (SATD) intended for medical purposes.^98^

It is essential to have an approximation between industry and academia to promote the validation of SATD, so that they may promote real benefits to the individual’s health.^98^ The development of apps and devices has a great future ahead, yet today they are still not as accurate as other traditional options and should be used with caution.

## FUTURE DIRECTIONS

Science and technology are continually advancing and together they can benefit therapists and patients in treating sleep disorders. Some future directions can be listed:

» The need to disseminate knowledge to lay people and health professionals about problems related to SDB and OSA, so that risk assessment can be performed more widely, and treatments can be conducted as early as possible, thus reducing the impact of negative consequences on the quality of life of individuals and society.
» Accomplishment of scientific studies and more tests to validate an increasing range of mobile apps and wearables related to SDB and OSA, allowing their use by patients more safely and reliably.
» Encourage the continuous evolution of the use of artificial intelligence and “machine learning” in the diagnosis and treatment of OSA.

## CONCLUSION

Obstructive sleep apnea is a problem that affects a great part of the population; if left untreated, it worsens the quality of life and poses serious consequences for the health of individuals. Orthodontists work in the region close to the upper airway and should be attentive to help identify patients with OSA and conduct specific treatment when indicated. New technologies are constantly emerging, associated with advances in artificial intelligence. Health professionals must be aware of this evolution to guide and interact properly with these new technologies, with patients with sleep disorders and other professionals involved in their treatment.
